# Supplementary material for: Fiber enhancement and 3D orientation analysis in label-free two-photon fluorescence microscopy
Source: Sci Rep. 2023 Mar 13;13:4160. doi: 10.1038/s41598-023-30953-w (PMC10011555; doi:10.1038/s41598-023-30953-w)
Supplement: Supplementary file 1 — Supplementary Information. [file 41598_2023_30953_MOESM1_ESM.pdf]

Supplementary Information

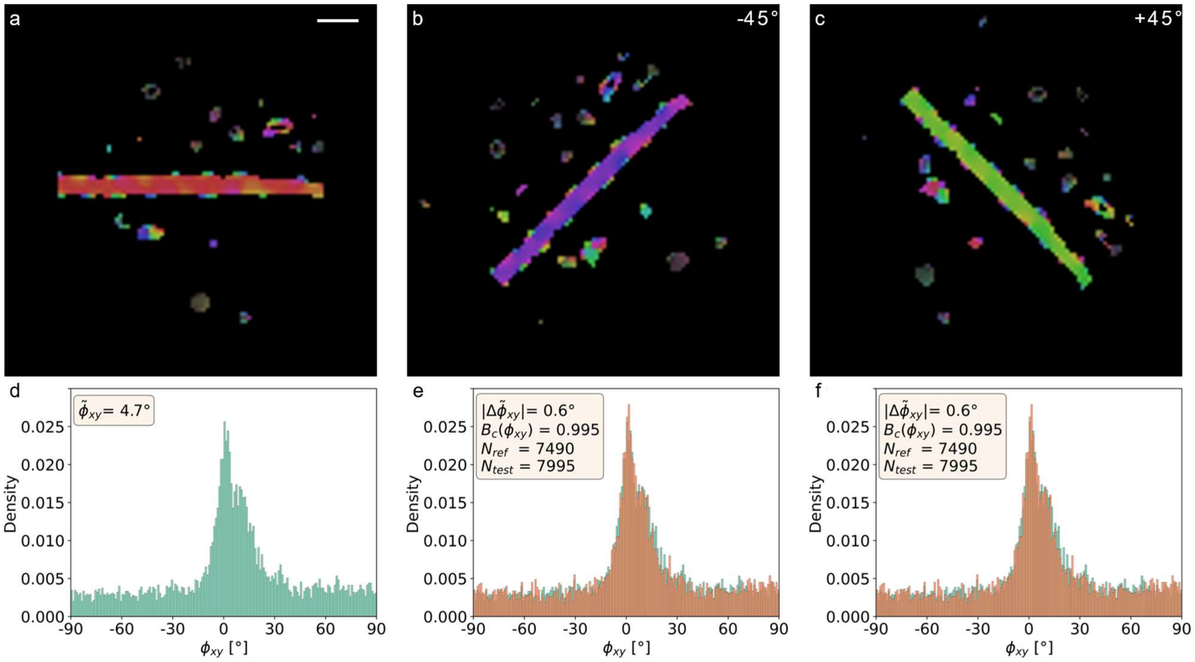

Figure S1 Example of the test rotations applied for validating the accuracy of the Frangi-based fiber orientation analysis pipeline (α = 0.001, β = 1, scales = [1, 1.25, 1.5] µm); the sliced 75 µm x 75 µm x 15 µm grey matter patch includes a myelinated fiber having an approximate diameter of ~5 µm: a) reference fiber orientation vectors, color-coded with respect to the in-plane azimuth angle φ (scale bar: 10 µm); b, c) orientation vectors obtained following 1D rotations of -45° and +45° about the z-axis; d) original distribution of the fiber azimuth angles estimated at a 1 µm x 1 µm x 1 µm voxel size; e, f) angular distributions generated from the rotated image patches (orange), and expressed in the adjusted spatial reference system.

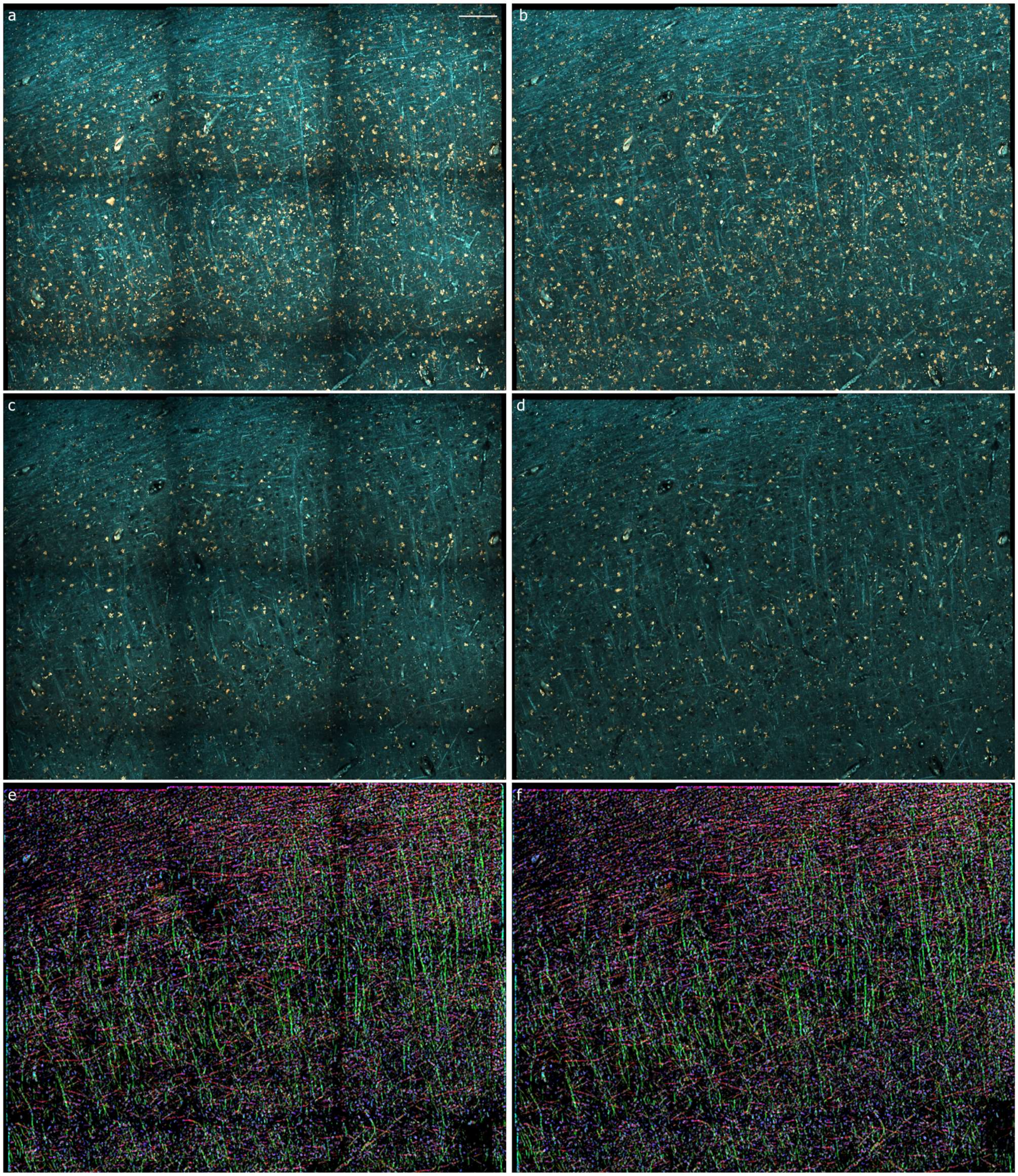

Figure S2 Effect of the CIDRE-based shading correction on the 3D fiber orientation maps returned by the Foa3D image processing pipeline. Whereas residual grid-like artefacts can be observed in the original maximum intensity projection (MIP), border discontinuities appear to be significantly reduced in the single plane images and the corresponding fiber orientation maps. a) MIP of a stitched TPFM tiled reconstruction (depth: 25 µm, scale bar: 100 µm); b) MIP following the uneven illumination correction; c) representative TPFM image at a depth of z = 5 µm; d) vignetting correction (z = 5 µm); e) average intensity projection (AIP) of the fiber orientation map resulting from the uncorrected tiled reconstruction; f) AIP of the fiber orientation map generated from the CIDRE-corrected tiles.

Table S1 Median ($q_{0.5}$) and quartiles ($q_{0.25,\,0.75}$) of the inter-median distance between the original reference distributions of the azimuth φ_{xy} and elevation θ_{zy} of myelinated fibers, and the (corrected) angular distributions generated following different 1D rotations around the z and x axes. The results related to grey (GM) and white matter (WM) image patches, randomly sampled from a test set of 3D TPFM tiles, are shown separately.

Tissue	Rotation [°]		-45	-40	-35	-30	-25	-20	-15	-10	-5	+5	+10	+15	+20	+25	+30	+35	+40	+45
$\Delta\tilde{\varphi}_{xy}$	GM N=250	$q_{0.75}$	2.6	2.5	2.4	2.2	1.8	1.5	1.2	1.0	0.7	0.8	0.9	1.2	1.6	1.8	2.1	2.4	2.6	2.6
		$q_{0.5}$	1.3	1.4	1.4	1.2	1.0	0.8	0.7	0.5	0.4	0.4	0.6	0.7	0.8	1.0	1.1	1.2	1.3	1.3
		$q_{0.25}$	0.6	0.7	0.6	0.6	0.6	0.4	0.3	0.3	0.2	0.2	0.3	0.4	0.4	0.4	0.5	0.6	0.6	0.6
	WM N=250	$q_{0.75}$	2.8	2.7	2.7	2.4	2.1	1.7	1.4	0.9	0.7	0.6	0.9	1.3	1.6	1.9	2.2	2.5	2.6	2.7
		$q_{0.5}$	1.9	1.8	1.8	1.7	1.5	1.3	1.0	0.6	0.4	0.4	0.6	0.8	1.1	1.3	1.4	1.7	1.8	1.9
		$q_{0.25}$	0.8	0.9	0.9	0.9	0.8	0.7	0.6	0.3	0.2	0.2	0.2	0.5	0.5	0.6	0.8	0.9	0.9	0.8
$\Delta\tilde{\theta}_{zy}$	GM N=250	$q_{0.75}$	5.3	5.8	6.0	5.4	5.1	4.4	3.6	3.0	1.7	1.7	2.3	3.2	4.0	4.4	4.6	4.7	4.3	3.7
		$q_{0.5}$	2.9	2.9	3.0	2.8	2.8	2.4	1.9	1.6	0.8	0.8	1.2	1.6	1.9	2.2	2.1	2.2	2.3	2.0
		$q_{0.25}$	1.5	1.5	1.5	1.3	1.2	1.2	0.9	0.7	0.4	0.4	0.6	0.7	0.8	0.8	1.0	1.0	1.1	1.0
	WM N=246	$q_{0.75}$	3,5	3.7	4.2	4.3	4.4	4.1	3.8	2.6	1.5	1.6	2.8	3.8	4.5	4.8	4.8	4.6	3.9	3.3
		$q_{0.5}$	2.2	2.4	2.6	2.7	2.8	2.6	2.2	1.5	0.8	0.8	1.5	2.4	2.7	2.9	2.9	2.7	2.6	2.2
		$q_{0.25}$	1.2	1.3	1.4	1.6	1.5	1.3	1.1	0.7	0.4	0.3	0.7	0.9	1.2	1.4	1.5	1.4	1.3	1.3

Table S2 Median ($q_{0.5}$) and quartiles ($q_{0.25,\,0.75}$) of the Bhattacharyya coefficient estimating the degree of overlap between the reference angular distributions of the azimuth φ_{xy} and elevation θ_{zy} of myelinated fibers, and the (corrected) angular distributions generated following different 1D rotations around the z and x axes. The results related to grey (GM) and white matter (WM) image patches, randomly sampled from a test set of 3D TPFM tiles, are shown separately.

Tissue	Rotation [°]		-45	-40	-35	-30	-25	-20	-15	-10	-5	+5	+10	+15	+20	+25	+30	+35	+40	+45
$B_c(\varphi_{xy})$	GM N=250	$q_{0.75}$	0.994	0.994	0.994	0.995	0.995	0.996	0.996	0.997	0.997	0.997	0.997	0.996	0.996	0.995	0.995	0.994	0.994	0.994
		$q_{0.5}$	0.993	0.993	0.993	0.994	0.994	0.995	0.995	0.996	0.996	0.996	0.996	0.995	0.995	0.994	0.994	0.993	0.993	0.993
		$q_{0.25}$	0.991	0.991	0.992	0.992	0.993	0.993	0.994	0.994	0.995	0.995	0.995	0.994	0.993	0.993	0.992	0.992	0.991	0.991
	WM N=250	$q_{0.75}$	0.994	0.994	0.994	0.995	0.995	0.996	0.996	0.997	0.997	0.997	0.997	0.996	0.996	0.995	0.995	0.995	0.994	0.994
		$q_{0.5}$	0.992	0.992	0.993	0.993	0.993	0.994	0.995	0.995	0.995	0.995	0.995	0.995	0.994	0.994	0.993	0.993	0.993	0.992
		$q_{0.25}$	0.990	0.989	0.990	0.990	0.990	0.991	0.991	0.992	0.992	0.992	0.992	0.991	0.991	0.991	0.990	0.990	0.989	0.989
$B_c(\theta_{zy})$	GM N=250	$q_{0.75}$	0.991	0.991	0.992	0.993	0.994	0.994	0.995	0.995	0.996	0.996	0.996	0.996	0.995	0.995	0.994	0.994	0.992	0.993
		$q_{0.5}$	0.986	0.987	0.988	0.989	0.991	0.992	0.994	0.994	0.995	0.995	0.994	0.994	0.994	0.993	0.992	0.991	0.990	0.991
		$q_{0.25}$	0.979	0.980	0.982	0.985	0.987	0.989	0.992	0.992	0.994	0.994	0.993	0.992	0.991	0.990	0.989	0.989	0.988	0.988
	WM N=246	$q_{0.75}$	0.988	0.988	0.990	0.992	0.993	0.994	0.995	0.995	0.996	0.996	0.996	0.996	0.995	0.995	0.995	0.995	0.994	0.994
		$q_{0.5}$	0.982	0.984	0.986	0.988	0.990	0.991	0.993	0.993	0.995	0.995	0.994	0.993	0.993	0.992	0.991	0.991	0.990	0.991
		$q_{0.25}$	0.974	0.975	0.980	0.983	0.986	0.988	0.991	0.991	0.992	0.992	0.991	0.991	0.989	0.988	0.987	0.987	0.985	0.986

Video S1 Example video of a 3D fiber orientation colormap (x-component: red channel; y-component: green channel; z-component: blue channel) generated from a 14 µm-deep TPFM section (isotropized pixel size; original uneven illumination).
